# Elderly people’s preferences for healthcare facilities in Shanghai: gender features and influencing factor analysis

**DOI:** 10.1186/s12889-023-15279-6

**Published:** 2023-02-17

**Authors:** Shangguang Yang, Luxue Liu, Chunlan Wang, Kevin Lo, Danyang Wang

**Affiliations:** 1 Economic Development Institute, East China University of Science and Technology, 200237 Shanghai, China; 2 Student Affairs Office, East China Normal University, 200062 Shanghai, China; 3 Population Research Institute, East China Normal University, 200062 Shanghai, China; 4 Department of Geography, Hong Kong Baptist University, 999077 Hong Kong, China

**Keywords:** Healthcare-seeking behavior, Elderly population, Influencing factor, China

## Abstract

**Background:**

China has one of the fastest paces of the growing aging population, High-level policymakers have recently recognized the aging population presents significant challenges to the Chinese healthcare system. In this context, the healthcare-seeking behaviors of the elderly population have become an essential field of study. It is necessary to understand their access to health services and to improve their quality of life, as well as to help policymakers to formulate healthcare policy. The study empirically investigates the factors influencing the elderly population’s healthcare-seeking behaviors in Shanghai, China, especially in choosing the quality of healthcare facilities to visit.

**Methods:**

We designed a cross-sectional study. The data of this study were derived from the “Shanghai elderly medical demand characteristics questionnaire” in the middle of November to early December 2017. A total of 625 individuals were included in the final sample. Logistic regression was adopted to investigate the differences in healthcare-seeking behaviors between elderly people when suffer from mild illness, severe illness and follow-up treatment. Next, the differences in gender were also discussed.

**Results:**

Factors affecting the healthcare-seeking behaviors of the elderly differ in mild illness and severe illness situations. For mild illness, demographic factors (gender and age) and socioeconomic factors (income and employment) play an important role in elderly healthcare choices. Female and older elderlies are more likely to choose local, lower-quality facilities, whereas those with high income and private employment are more likely to choose higher-quality facilities. For severe illness, socioeconomic factors (income and employment) are important. Furthermore, individuals with basic medical insurance are more likely to choose lower-quality facilities.

**Conclusion:**

This study has shown that the affordability of public health services should be addressed. Medical policy support may be an important way to reduce the gap in access to medical services. We should pay attention to the gender differences in the elderly’s choice of medical treatment behavior, consider the differences in the needs of male and female elderly. our findings are only for elderly Chinese participants in the greater Shanghai area.

## Background

The world is undergoing an unprecedented demographic shift. Driven by increasing life expectancy and declining birth rates, many countries are experiencing population aging [1, 2]. China has one of the fastest paces of the growing aging population [3–6]. The proportion of the over-65 population surged from 5.6% to 1990 to 8.87% in 2010 [7, 8]. In 2019, the over-60 population reached 253.88 million, accounting for 18.1% of the country’s total population (National Bureau of Statistics of China, 2020). It is predicted that, by 2050, there will be approximately 400 million people over 65 years old in China, or one in every four [9, 10]. The aging population presents significant challenges to the Chinese healthcare system [11, 12]. High-level policymakers have recently recognized this pressing problem. In 2016, the State Council promulgated the “Outline of Healthy China 2030 Plan”, aiming to improve public health services and narrow the gap in the provision of healthcare between urban and rural areas and different age groups. The 19th National Congress of the Communist Party of China also featured a “Healthy China Strategy” to tackle population aging.

In this context, the healthcare-seeking behaviors of the elderly population have become an essential field of study. Healthcare-seeking behaviors refer to individuals’ choice of health services to meet their health demands, including selecting healthcare providers [13]. As the increasing proportion of aging populations presents new challenges for health policymakers and professionals [14], studying healthcare-seeking behaviors of the elderly becomes necessary to understand their access to health services and to improve their quality of life, as well as to help policymakers to formulate healthcare policy [15–17]. In particular, understanding elderly healthcare-seeking behaviors is crucial for improving healthcare coverage and the efficiency of the national healthcare system.

Demographic characteristics such as gender, age, marital status, residence status and family income will greatly affect the medical choice of elderly people [18–20]. For example, the study believes that women living in cities and with higher education are likely to choose primary medical institutions for medical treatment [21].In rural areas of China, elderly with low income may choose to doctor themselves [22]. Marriage can promote young people to form healthy behaviors and have a lasting impact. Compared with women, marriage is more beneficial to men [23]. In Vietnam, it is reported that parents pay more attention to boys in the process of their children’s health care, and gender inequality will have a negative impact on the health of girls [24]. Women tend to conduct self-evaluation, so women are more likely to use preventive medical services more frequently than men [18, 25–27]. But some women had fewer economic resources, women are less likely to go to hospital than men with similar health conditions [28].

Social and economic characteristics such as income, education, employment, family economic support and medical insurance also affect the elderly’s choice of medical treatment [29–31]. Compared with the lower income group, people with high income have a stronger ability to resist the economic risk of disease at the moment. groups with low income are vulnerable to the economic risks of diseases. in China, participation in the new rural cooperative medical plan has significantly increased the probability of elderly people going to rural hospitals [22]. People with higher education will choose to seek medical treatment in time [32].

Third, health status may influence individuals’ decisions on healthcare services. For example, chronic illness and disability status have a positive impact on the use of healthcare services [33].

Fourth, provider-side factors, such as, transportation availability, medical expenses, medical insurance and the perceived quality of healthcare services, are predictors of healthcare-seeking behaviors [34, 35]. For example, in China, medical insurance is an auxiliary measure for elderly patients. Elderly with medical insurance shall go to designated medical institutions for medical treatment with medical insurance certificate. Designated medical institutions or designated retail pharmacies can deduct, which is settled directly with basic medical insurance funds. If the elderly has fixed job before retirement, their medical insurance is Urban Employees Basic Medical Insurance, their reimbursement level within the scope of medical insurance reached about 85%. If the elderly not have a formal job, they can use Urban Resident Medical Insurance. They can choose different hospitals according to the proportion of reimbursement, The higher the hospital level, the less the reimbursement proportion.

Drawing on an empirical study from Shanghai, this quantitative study aims to examine the factors influencing the elderly population’s healthcare-seeking behaviors, especially in choosing the quality of healthcare facilities to visit. Shanghai is an ideal case study because population aging in China and globally is primarily an urban phenomenon [36]. The urban focus of healthy aging is underscored by publications, including the Global Age-Friendly Cities Guide (World Health Organization) and Checklist of Essential Features of Age-Friendly Cities [37]. The concentration of elderly in Chinese mega-cities is exceptionally high [36, 38]. As a leading metropolis in China, Shanghai has one of the largest elderly populations in the country—Shanghai’s over-60 population had already reached 4.58 million (31.6% of the population) by the end of 2016 [39]. The results give evidence-based support to the municipal governments of Chinese cities in the provision of public healthcare service and enrich the literature by presenting an example anchored within a Chinese context.

## Methods

Survey subjects.

1. The research was conducted in the middle of November to the first ten days of December 2017;
2. Two streets were randomly selected from each district, and 30 people were randomly selected from each street as the respondents;
3. The respondents were permanent residents aged 60 years and above in Shanghai, mainly those with registered residence. Through multi-stage stratified random sampling, we randomly selected:

- A number of districts in the city’s central area, suburbs, and outer suburban areas; then.
- Two streets in each district; and then.
- 30 people were randomly selected from neighbourhood committees in each street as the respondents.

4. On-site and face-to-face interview was mainly used to collected questionnaires, filling in and taking back on site. Before the formal survey, we conducted a small-scale pilot survey in three districts to ensure that the questionnaire design was reasonable. The formal survey was based on modifications of the initial one.

The survey covered 12 districts (Jing’an, Huangpu, Xuhui, Putuo, Yangpu, Changning, Minhang, Pudong New Area, Baoshan, Jiading, Songjiang, and Fengxian), covering 39 streets (in towns) and 138 residential committees (132 urban committees and 6 village committees), The “residential committee” is an important basis for the grass-roots political power in China’s cities, and also one of the bridges and links between the government and the people, the main function is to provide basic services for residents. These districts encompassed the central urban area, suburbs area, outer suburban areas. The respondents covered the areas divided by Shanghai’s inner ring, middle ring, and outer ring (these districts include Jing’an, Huangpu, and Hongkou in the urban core areas; Xuhui, Changning, Putuo, and Yangpu in the urban fringes; Pudong, Minhang, Baoshan, and Jiading in the suburbs; and Jinshan, Songjiang, Fengxian, Qingpu, and Chongming in the outer suburban areas. the urban core areas and urban fringes is central urban area).

The survey takers were undergraduates of the Economic Development Institute of East China University of Science and Technology who had undergone uniform and strict training. We communicated with each elderly individual alone and told him/her that we would keep the conversation confidential. The questionnaire survey was conducted after obtaining verbal “informed consent” from the elderly individual.

### Study design

This research is based on a cross-sectional design. The data were analyzed by SPSS using descriptive statistics and regression. The analysis considers the differences in healthcare-seeking behaviors between elderly people with mild and severe illnesses, as it has been suggested that differences in healthcare demands, including health condition and disease severity, may play a role in determining individuals’ healthcare-seeking behaviors [40, 41]. For example, individuals with a serious illness or injury may prefer treatment in a higher-quality health facility. In contrast, individuals with minor illnesses or injuries may prefer lower-quality, less expensive health facilities [42].

Hospitals in China were classified according to a 3-tier system that represents a hospital’s ability to provide medical care, medical education, and conduct medical research. Hospitals were labelled as level 1, level 2, or level 3. A hospital labelled as level 1 is typically contains less than 100 beds. Often named community clinics, they are tasked with providing preventative care, minimal health care, and rehabilitation services. Level 2 hospitals are usually named district hospital, they tend to be affiliated with a medium size county or district and contain more than 100 beds but less than 500. They are responsible for providing comprehensive health services as well as medical education and conducting research on a regional basis. Level 3 hospitals are usually named general hospital which have a bed capacity exceeding 500, they are capable of providing specialist health services and medical education as well as scientific research, and thus they serve as medical hubs providing care to multiple regions.

### Variables and tools

The dependent variable of the study is the quality of healthcare services the elderly prefers when suffering from an illness. Healthcare quality is generally measured by the quality of the medical institution, availability of medical professionals, quality of medical resources, medical level, availability of prescription medicine in a medical facility, and environment for medical treatment or hospitalization [43–45]. In this study, we measured the healthcare quality of medical institutions by hospital grade. In China, healthcare quality can be measured in terms of access to two major groups of medical institutions: tertiary and non-tertiary hospitals. According to the three-tier grading system for Chinese hospitals, tertiary hospitals provide high-quality, specialized medical services, secondary hospitals are regional facilities that serve the local population, and primary hospitals are basic healthcare units such as village health stations, township health centers, outpatient clinics, and community health centers [46]. According to an official report, China has 2749 general hospital (Level 3), 9687 district hospital (Level 2), and 11,264 community clinics (Level 1). In this study, we use “whether an individual chooses a tertiary hospital” as the dependent variable in the model analysis, with a value of 1 when a tertiary hospital is chosen and a value of 0 otherwise.

Selected 12 independent variables representing the four dimensions: gender, age, marital status, and cohabitation status for demographic characteristics; education, occupation before retirement, monthly income, household registration, and place of residence for socioeconomic characteristics; physical condition and chronic diseases for state of health; and basic medical insurance for medical security status.

The factors indicating the elderly population’s preferences for healthcare services are compared according to healthcare demand: mild illness or severe illness. We define mild illness as relatively common diseases such as fevers, colds, migraines, and diarrhea. Severe illness refers to serious, long-term diseases that decrease the patient’s quality of life and incur high healthcare expenditures. Such diseases include malignant tumors, severe brain injuries, severe cardiovascular and cerebrovascular diseases, deep comas, advanced chronic diseases, permanent paralysis, lifelong disabilities, severe Parkinson’s disease, and severe mental illness.

Used logistic regression to analyze the effects of the 12 independent variables on the elderly population’s healthcare preference in the cases of individuals suffering from mild illness or severe illness with long-term treatment. The healthcare-seeking behaviors model for the elderly is specified as follows: Y = 1: choosing a general hospital (Level 3) for medical treatment, Y = 0: choosing a non-general hospital for medical treatment. $\text{P}(\text{y}=1\left|{\text{x}}_{\text{i}}\right)={\text{p}}_{\text{i}}$ represents the probability that an elderly individual chooses a tertiary hospital for medical treatment, and the *k* independent variables that may affect the dependent variable Y are denoted as ${\text{x}}_{1},{\text{x}}_{2},{\text{x}}_{3},\dots ,{\text{x}}_{\text{k}}$.

## Results

The questionnaires were administered face-to-face to 638 participants. After excluding the incomplete responses, 625 valid questionnaires were included in the sample, with 224 from men and 401 from women. Some of elderly people interviewed did not answer the key variables in regression, so the sample size used in the regression was 611.

### Characteristics of respondents

The questionnaire items were divided into the following five sections: sociodemographic characteristics, health conditions, healthcare preferences, expectations and satisfaction with healthcare services, and possession of medical insurance. Characteristics of study respondents is shown in Table 1.

**Table 1:** Characteristics of study respondents

Characteristics			N (%)
Demographic factors	Gender	Male Female	224 (35.84) 401 (64.16)
	Age	60–69 70–79 80 and above	377 (60.32)
			186 (29.76)
			62 (9.92)
	Marital status	Unmarried Married Divorce/widow	11 (1.76)
			505 (80.8)
			109 (17.44)
	Cohabitation status	Living alone Couple living together Live with relatives Live with children Live with three generations	56 (8.96)
			307 (49.12)
			21 (3.36)
			169 (27.04)
			72 (11.52)
Socioeconomic factors	Education	Elementary school and below Junior high school (technical) secondary school College and above	131 (20.96)
			266 (42.56)
			150 (24.00)
			78 (12.48)
	Occupation before retirement	Unemployed Government institutions Collective enterprises Private and others	76 (12.16)
			400 (64.00)
			90 (14.40)
			59 (9.44)
	Monthly income (yuan)	2000 and below 2000–3999 4000–5999 6000 and above	121 (19.80)
			303 (49.59)
			145 (23.73)
			41 (6.71)
	Household registration	Non-Shanghai hukou Shanghai hukou	30 (4.80)
			595 (95.20)
	Place of residence	Rural Urban	555 (88.80)
			70 (11.20)
State of health	Physical condition	Unhealthy Moderately healthy Healthy	82 (13.12)
			267 (42.72)
			376 (60.16)
	Chronic diseases	No One	186 (29.76)
			253(41.40)
		Two	128(20.94)
		Three or more	58 (9.49)
Medical security	Basic medical insurance	Not participated Participated	43 (6.88)
			582 (93.12)

**Fig. 1:**
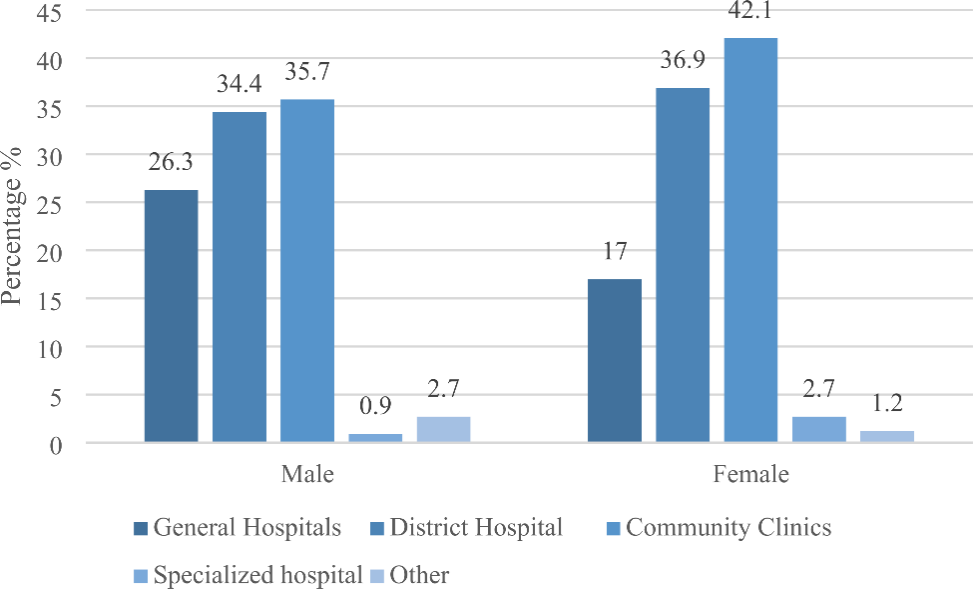
Gender differences in medical seeking behavior of the elderly with mild illness

When suffering from mild illness, male elderly people are more likely to go to higher level hospitals than female elderly people. The survey shows that when the elderly suffering from mild illness, the proportion of choosing community health service centers for medical treatment is the highest, 39.8%, followed by district hospitals (Level 2), accounting for 36.0%, while the proportion of choosing general hospitals (Level 3) is 20.3%, and the proportion of specialty hospitals and others is 3.9%. Among them, 26.3% of the male elderly choose to go to the general hospital (Level 3) when suffering from mild illness, which is 9.3% points higher than that of the female elderly; The proportion of male elderly people who choose to go to district center or district hospital (level 2) and community health service center is 34.4% and 35.7% respectively, which is 2.5% points and 6.4% points lower than that of female elderly people. However, the proportion of male and female elderly people choosing to go to specialized hospitals and other ways is similar (Fig. 1).

**Fig. 2:**
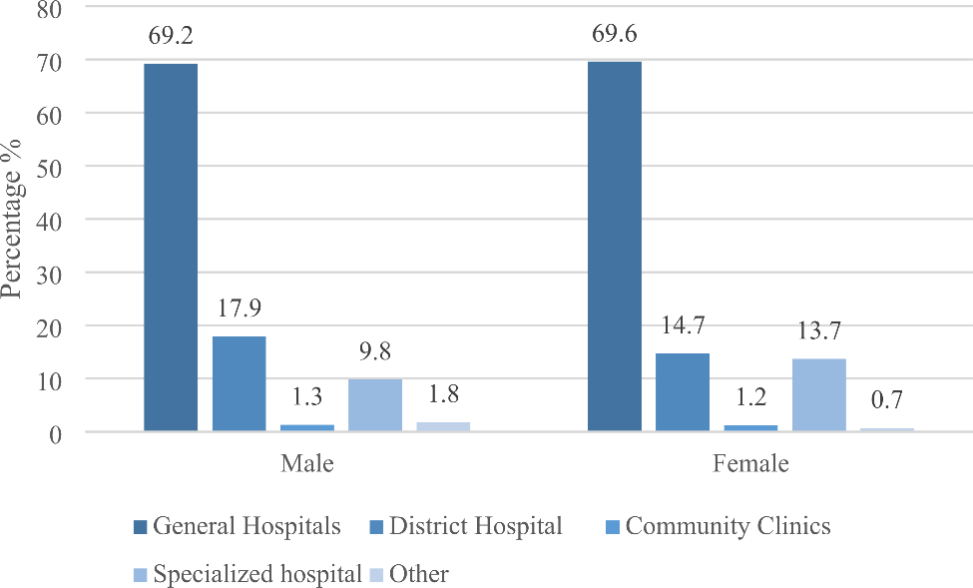
Gender differences in medical seeking behavior of the elderly with severe illness

When suffering from severe illness, the elderly is more likely to go to higher-level hospitals for medical treatment. But there are some gender differences, when suffering from severe illness, both male and female elderly people are more inclined to go to hospitals with higher grades, among which the proportion of female elderly people choosing general hospitals (Level 3) is 69.6%, only 0.4% points higher than male elderly people, which is consistent with our expectations. When faced with major diseases that may threaten life safety, both male and female elderly people will be more active in choosing medical treatment. The proportion of male elderly people who choose to go to district hospital (Level 2) for medical treatment is 17.9%, 3.2% points higher than that of female elderly people. In addition, female elderly people prefer specialized hospitals when suffering from major diseases, accounting for 13.7%, 3.9% points higher than male elderly people (Fig. 2).

**Fig. 3:**
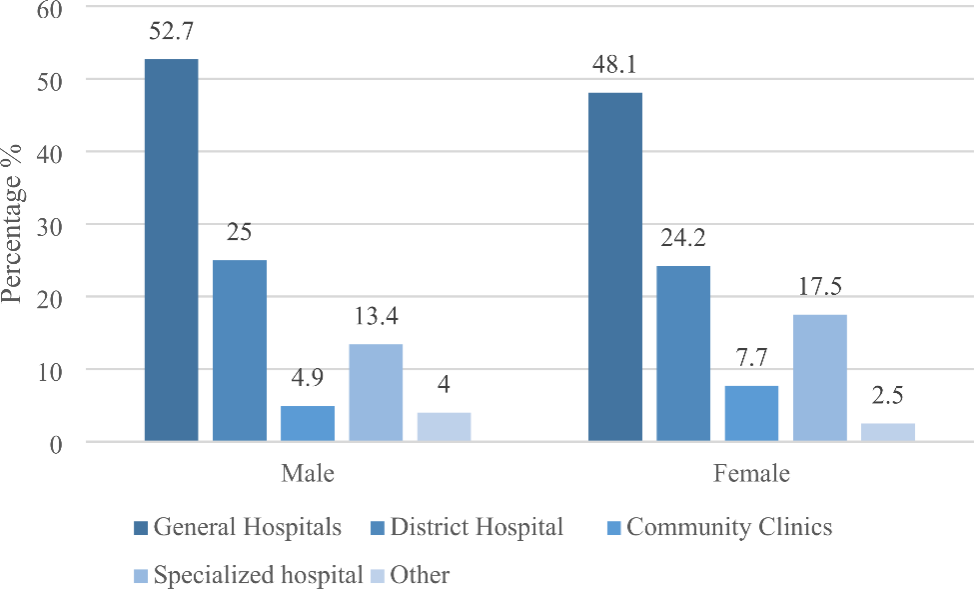
Gender differences in medical seeking behavior of the elderly with follow-up treatment

For follow-up treatment of severe illness, male elderly people are more likely to go to higher-level hospitals than female elderly people, while the proportion of female elderly people choosing to go to community health service centers and specialized hospitals is slightly higher than male elderly people. More than half of the male elderly still choose the general hospital (level 3) for treatment when suffering from major diseases, and the corresponding proportion is 4.6% points higher than the female elderly. Some studies have shown that men are more prone to fatal diseases, such as stroke, chronic lung disease and other occupational-related diseases. Men play more of the role of “tool man” in companies and families. Due to long-term investment in the labor market, their health loss is also relatively serious. The proportion of male elderly people who choose to go to district hospital (level 2) for medical treatment is 25.0%, only 0.8% points higher than that of female elderly people. In addition, female elderly people prefer specialized hospitals when suffering from major diseases, accounting for 17.5%, 4.1% points higher than male elderly people (Fig. 3).

### Regression analysis

Table 2 shows the regression results of the elderly’s medical behavior when they experience mild illness. For Full sample, it can be seen that gender, age, occupation before retirement, monthly income, place of residence has a significant impact on the elderly’s medical behavior. Specifically, for male sample, compared with the unemployed, Men who worked in private enterprises before retirement (OR = 3.90 95%CI :1.17,12.98) are more likely to go to the general hospital (Level 3). Compared with the unhealthy, Men who moderately healthy (OR = 0.34 95%CI:0.11,1.04) are more likely to go to the general hospital (Level 3). For female sample, compared with the 60–69 years-old people, 70–79 years old (OR = 0.37 95%CI:0.17,0.81) are more likely to choose the Level 3. Compared with the unemployed, Women who worked in governmental institutions (OR = 0.29 95%CI:0.08,1.15) are more likely to choose the Level 3. Compared with the unhealthy, healthy female (OR = 2.51 95%CI:0.87,7.21) are more likely to choose the Level 3. Compared with women living in rural areas, place of residence in urban (OR = 12.56 95%CI:1.34,117.98) are more likely to choose the Level 3 (Table 2).

**Table 2:** Logistic regression results of the elderly with mild illness

Variable	Full sample		Male		Female	
	(1)		(2)		(3)	
	OR	(95% CI)	OR	(95% CI)	OR	(95% CI)
**Gender(reference**: Male**)**			-	-	-	-
female	-0.57**	(0.34,0.95)	-	-	-	-
**Age group (reference: 60–69 years-old)**						
70–79	0.43***	(0.25,0.74)	0.58	(0.26,1.28)	0.37**	(0.17,0.81)
80 and above	0.89	(0.42,1.89)	1.42	(0.49,4.14)	0.63	(0.2,2.00)
**Marital status (reference: single)**						
Married with a spouse	0.62	(0.12,3.26)	0.57	(0.05,6.08)	0.54	(0.04,7.45)
Divorce/widow	1.81	(0.32,10.27)	0.76	(0.05,10.62)	2.02	(0.14,28.26)
**Cohabitation status (reference: living alone)**						
Couple living together	2.27	(0.83,6.24)	4.65	(0.41,52.70)	2.06	(0.58,7.29)
Live with relatives	2.92	(0.68,12.52)	5.51	(0.31,96.43)	2.75	(0.42,18.09)
Live with children	1.81	(0.72,4.50)	4.00	(0.37,42.66)	1.49	(0.52,4.31)
Live with three generations	1.27	(0.40,4.00)	3.57	(0.28,45.75)	0.99	(0.22,4.41)
**Education level (reference: elementary school and below)**						
Junior high school	1.47	(0.72,3.01)	1.53	(0.49,4.77)	1.36	(0.51,3.67)
Secondary school	1.85	(0.84,4.03)	2.02	(0.59,7.00)	1.68	(0.56,5.02)
College and above	1.37	(0.54,3.43)	1.68	(0.43,6.50)	0.97	(0.23,4.16)
**Occupation before retirement (reference: unemployed)**						
Governmental institutions	0.35**	(0.13,0.92)	0.46	(0.10,2.14)	0.29*	(0.08,1.15)
Collective enterprises	1.15	(0.61,2.18)	0.71	(0.18,2.83)	1.35	(0.63,2.88)
Private and others	2.67**	(1.16,6.13)	3.90**	(1.17,12.98)	2.01	(0.53,7.63)
**Monthly income (reference: less than 2,000 yuan)**						
2000–3999	1.90	(0.75,4.83)	1.03	(0.22,4.83)	2.11	(0.59,7.47)
4000–5999	2.46*	(0.87,6.92)	1.83	(0.39,8.52)	2.81	(0.61,12.9)
6000 and above	3.87**	(1.12,13.39)	2.99	(0.48,18.56)	3.80	(0.51,28.36)
**Physical condition (reference: unhealthy)**						
Moderately healthy	0.73	(0.35,1.51)	0.34*	(0.11,1.04)	1.22	(0.43,3.46)
Healthy	1.46	(0.70,3.04)	0.57	(0.18,1.83)	2.51*	(0.87,7.21)
**Chronic disease (reference: none)**						
One	0.89	(0.53,1.50)	0.88	(0.39,1.99)	0.88	(0.42,1.85)
Two	1.59	(0.85,2.97)	1.18	(0.40,3.45)	1.78	(0.79,4.05)
Three or more	1.20	(0.50,2.86)	0.93	(0.24,3.57)	1.64	(0.47,5.77)
**Basic medical insurance (reference: not participated)**						
Participated	1.58	(0.57,4.41)	2.49	(0.27,22.82)	1.33	(0.39,4.54)
**Place of residence (reference: rural)**						
urban	4.63**	(1.37,15.68)	2.39	(0.46,12.36)	12.56**	(1.34,117.98)
Household registration (reference: non-Shanghai hukou)						
Shanghai hukou	1.62	(0.52,5.11)	1.50	(0.30,7.43)	1.51	(0.27,8.46)
Constant	0.23***	(0.00,0.29)	0.03	(0.00,2.34)	0.01***	(0.00,0.26)
R^2^	0.11		0.11		0.13	
N	611		215		396	

*p < 0.05, **p < 0.01

Table 3 shows the regression results of the elderly’s medical behavior when they experience severe illness. For full sample, physical condition has a significant impact on the elderly’s medical behavior. From the perspective of gender differences, for male, compared with single, Couple living together (OR = 5.25 95%CI:0.77,35.76) and Live with three generations (OR = 18.48 95%CI:1.94,175.73) are more likely to go to the general hospital (Level 3). Male elderly people who have attended junior high school (OR = 2.53 95%CI:0.90,7.14) are more likely to choose general hospital than those with lower education. Monthly income. Male elderly with a monthly income of more than 6000 yuan (OR = 10.5 95%CI: 1.52,72.69) are more likely to choose general hospital than male elderly with a monthly income of less than 2000 yuan. Men who moderately healthy (OR = 0.33 95%CI: 0.09,1.20) are more likely to go to the general hospital (Level 3). For female sample, compared with the 60–69 years-old people, 70–79 years old (OR = 0.56 95%CI: 0.33,0.96) are more likely to choose the Level 3. Female elderly people with Shanghai hukou (OR = 0.15 95%CI: 0.02,1.25) are more likely to choose the Level 3(Table 3).

**Table 3:** Logistic regression analysis of the elderly with severe illness

Variable	Full sample		Male		Female	
	(1)		(2)		(3)	
	OR	(95% CI)	OR	(95% CI)	OR	(95% CI)
**Gender(reference: Male)**						
Female	1.13	(0.73,1.75)	-	-	-	-
**Age group (reference: 60–69 years-old)**						
70–79	0.72	(0.47,1.09)	1.14	(0.55,2.37)	0.56**	(0.33,0.96)
80 and above	1.15	(0.57,2.33)	1.04	(0.32,3.32)	1.35	(0.49,3.69)
**Marital status (reference: single)**						
Married with a spouse	0.80	(0.18,3.48)	0.17	(0.01,2.99)	1.86	(0.28,12.35)
Divorce/widow	0.87	(0.19,4.03)	0.39	(0.02,9.20)	1.57	(0.23,10.64)
**Cohabitation status (reference: living alone)**						
Couple living together	0.83	(0.34,2.04)	5.25*	(0.77,35.76)	0.51	(0.17,1.53)
Live with relatives	0.61	(0.17,2.23)	1.03	(0.07,14.13)	0.42	(0.08,2.08)
Live with children	0.75	(0.33,1.72)	4.06	(0.63,25.99)	0.48	(0.18,1.26)
Live with three generations	1.56	(0.56,4.31)	18.48**	(1.94,175.73)	0.85	(0.26,2.83)
**Education level (reference: elementary school and below)**						
Junior high school	1.34	(0.79,2.29)	2.53*	(0.90,7.14)	1.15	(0.58,2.27)
Secondary school	1.23	(0.66,2.28)	1.45	(0.46,4.57)	1.30	(0.58,2.91)
College and above	1.16	(0.55,2.44)	0.84	(0.25,2.81)	1.99	(0.65,6.06)
**Occupation before retirement (reference: unemployed)**						
Governmental institutions	1.04	(0.47,2.27)	1.47	(0.35,6.08)	0.82	(0.28,2.38)
Collective enterprises	0.81	(0.48,1.38)	0.5	(0.17,1.49)	0.84	(0.44,1.59)
Private and others	0.91	(0.44,1.87)	1.15	(0.34,3.89)	0.67	(0.24,1.84)
**Monthly income (reference: less than 2,000 yuan)**						
2000–3999	1.30	(0.63,2.67)	1.67	(0.44,6.27)	1.08	(0.42,2.77)
4000–5999	1.53	(0.67,3.49)	2.72	(0.70,10.56)	1.05	(0.32,3.47)
6000 and above	2.34	(0.77,7.14)	10.5**	(1.52,72.69)	0.54	(0.11,2.67)
**Physical condition (reference: unhealthy)**						
Moderately healthy	0.52**	(0.27,1.00)	0.33*	(0.09,1.20)	0.54	(0.25,1.19)
Healthy	0.51**	(0.26,0.99)	0.34	(0.09,1.29)	0.52	(0.23,1.17)
**Chronic disease (reference: none)**						
One	1.10	(0.71,1.69)	1.75	(0.80,3.86)	0.97	(0.55,1.71)
Two	1.38	(0.80,2.39)	1.03	(0.37,2.86)	1.69	(0.84,3.40)
Three or more	1.13	(0.54,2.35)	1.85	(0.48,7.11)	1.17	(0.46,3.02)
**Basic medical insurance (reference: not participated)**						
Participated	0.74	(0.32,1.70)	0.58	(0.12,2.71)	0.87	(0.31,2.46)
**Place of residence (reference: rural)**						
urban	1.10	(0.54,2.24)	0.76	(0.20,2.89)	1.59	(0.62,4.08)
**Household registration (reference: non-Shanghai hukou)**						
Shanghai hukou	0.69	(0.25,1.87)	2.24	(0.51,9.73)	0.15*	(0.02,1.25)
Constant	5.81	(0.68,49.42)	1.06	(0.02,68.58)	20.62*	(0.95,446.95)
R^2^	0.04		0.13		0.05	
N	611		215		396	

*p < 0.05, **p < 0.01

Table 4 shows the regression results of the elderly’s medical behavior when they need follow-up treatment. From the results of the whole sample, marital status, occupation before retirement, monthly income, basic medical insurance play important role on the elderly decision. For male, married (OR = 0.04 95% CI: 0.00,0.90) is more likely to go to general hospital than single. Live with three generations (OR = 5.27 95% CI: 0.82,33.84) is more likely to go to general hospital. Male elderly with a monthly income of 2000–3999 yuan (OR = 3.20 95%CI: 0.81,12.67) and 4000–5999(OR = 3.44 95%CI: 0.85,13.94) are more likely to choose general hospital than male elderly with a monthly income of less than 2000 yuan. Compared with women living in rural areas, place of residence in urban (OR = 0.24 95%CI: 0.06,0.91) are more likely to choose the Level 3. For women, lived with relatives (OR = 0.25 95%CI: 0.05,1.30) are more likely to choose the Level 3. Women with higher incomes and participating in basic medical insurance (OR = 0.43 95%CI: 0.17,1.09) are more likely to go to general hospital (Table 4).

**Table 4:** Logistic regression analysis of the elderly with follow-up treatment

Variable	Full sample		Male		Female	
	(1)		(2)		(3)	
	OR	(95% CI)	OR	(95% CI)	OR	(95% CI)
**Gender(reference: Male)**						
Female	0.90	(0.60,1.35)	-	-	-	-
**Age group (reference: 60–69 years-old)**						
70–79	1.03	(0.69,1.52)	0.77	(0.39,1.51)	1.17	(0.70,1.94)
80 and above	1.10	(0.59,2.06)	0.92	(0.34,2.5)	1.12	(0.47,2.70)
**Marital status (reference: single)**						
Married with a spouse	0.30*	(0.07,1.24)	0.04**	(0.00,0.90)	0.64	(0.1,3.95)
Divorce/widow	0.43	(0.10,1.87)	0.10	(0.00,2.90)	0.68	(0.11,4.25)
**Cohabitation status (reference: living alone)**						
Couple living together	0.90	(0.40,2.04)	2.00	(0.35,11.46)	0.64	(0.24,1.71)
Live with relatives	0.33	(0.09,1.24)	0.25	(0.02,3.91)	0.25*	(0.05,1.30)
Live with children	1.07	(0.51,2.25)	3.00	(0.54,16.51)	0.77	(0.33,1.83)
Live with three generations	1.37	(0.57,3.33)	5.27*	(0.82,33.84)	0.87	(0.30,2.54)
**Education level (reference: elementary school and below)**						
Junior high school	1.00	(0.60,1.66)	1.64	(0.61,4.41)	0.73	(0.38,1.41)
Secondary school	0.86	(0.48,1.54)	0.87	(0.29,2.58)	0.82	(0.38,1.76)
College and above	0.76	(0.38,1.54)	1.42	(0.44,4.57)	0.52	(0.19,1.42)
**Occupation before retirement (reference: unemployed)**						
Governmental institutions	0.72	(0.34,1.55)	0.48	(0.12,2.01)	0.69	(0.25,1.92)
Collective enterprises	1.46	(0.88,2.41)	0.95	(0.34,2.66)	1.51	(0.83,2.75)
Private and others	1.98*	(0.97,4.04)	2.53	(0.75,8.60)	1.35	(0.50,3.61)
**Monthly income (reference: less than 2,000 yuan)**						
2000–3999	2.89***	(1.39,6.00)	3.20*	(0.81,12.67)	2.76**	(1.08,7.03)
4000–5999	3.48***	(1.53,7.92)	3.44*	(0.85,13.94)	3.97**	(1.25,12.60)
6000 and above	4.75***	(1.69,13.3)	4.05	(0.75,21.88)	6.06**	(1.28,28.63)
**Physical condition (reference: unhealthy)**						
Moderately healthy	0.8	(0.46,1.40)	1.03	(0.36,2.95)	0.72	(0.36,1.42)
Healthy	0.98	(0.55,1.75)	1.87	(0.61,5.75)	0.7	(0.35,1.43)
**Chronic disease (reference: none)**						
One	0.88	(0.59,1.33)	0.56	(0.27,1.16)	1.09	(0.64,1.86)
Two	1.25	(0.75,2.07)	1.89	(0.71,5.03)	1.15	(0.61,2.15)
Three or more	0.86	(0.43,1.69)	1.03	(0.31,3.39)	0.94	(0.39,2.25)
**Basic medical insurance (reference: not participated)**						
Participated	0.52*	(0.25,1.10)	0.59	(0.16,2.26)	0.43*	(0.17,1.09)
**Place of residence (reference: rural)**						
Urban	0.77	(0.38,1.55)	0.24**	(0.06,0.91)	1.35	(0.54,3.37)
**Household registration (reference: non-Shanghai hukou)**						
Shanghai hukou	0.52	(0.21,1.28)	0.76	(0.18,3.10)	0.37	(0.10,1.36)
Constant	7.70**	(1.08,54.75)	40.32*	(0.59,2734.59)	6.9	(0.57,83.81)
R2	0.05		0.11		0.06	
N	611		215		396	

*p < 0.05, **p < 0.01

## Discussion

There is no evidence suggest that “high quality” hospitals have better outcomes than “low quality” hospitals for the same conditions, particularly mild illness. The statistics also show that when the elderly suffer from mild illness, the proportion of choosing community health service centers for medical treatment is the highest, but the male elderly is more inclined to go to higher-level hospitals than the female elderly. When suffering from severe illness, the elderly all more likely to go to higher-level hospitals for medical treatment, gender differences are almost non-existent. For follow-up treatment of major diseases, male elderly people are more likely to go to higher-level hospitals than female elderly, while the proportion of female elderly people choosing to go to community health service centers and specialized hospitals is slightly higher than male elderly people. The reasons for this phenomenon may be that in Shanghai, the megacity of China, patients do not trust basic medical services, basic hospital services are imperfect, and the design of hospital referral system is unreasonable.

Medical seeking behavior is a person’s medical seeking decision based on comprehensive consideration of comprehensive physical condition, economic affordability, accessibility of medical resources, cognition of disease, psychological expectation, family support, social environment, etc. It is a complex and systematic process. The results of this paper show that, in case of mild illness, for men, occupation before retirement, physical condition and monthly income are important factors affecting the choice of medical treatment, while for women, age, occupation before retirement, physical health and residence are important. When seriously ill, for men, residential relationship, education level, monthly income and health status are important. For women, age and registered residence status are particularly important. When follow-up treatment is needed, for men, marital status, living status, monthly income and living place are important. For women, living conditions, monthly income and participation in basic medical insurance are important.

The differences might be influenced by the conventional male’s role as the “backbone” of the family. The gender differences may also be affected by the “male outside, female inside” tradition, in which females are stereotypically responsible for housework and do not play important decision-making roles. Moreover, in most Chinese families, the female elderly played an important role in caring for family members. Thus, they might have less spare time for their personal lives, which may explain why the female elderly tend to choose non-tertiary hospitals for medical treatment when suffering from mild illnesses. However, this gender difference diminishes among the elderly suffering from severe diseases with long-term treatment. A possible explanation is that individuals consider staying alive to be critical, regardless of gender.

The level of income by-and-large determines the elderly’s medical service quality and ability to pay. Our results show that income has a significant impact on male elderly’s healthcare-seeking behaviors: those with higher income are more likely to choose general hospital in both the case of mild and severe illness. Occupation before retirement is an important factor affecting an individual’s social and economic status. Different occupations imply various types of medical security, which, in turn, affect the healthcare-seeking behaviors of the elderly. Not surprisingly, those elderly who had worked in the high company benefits, which typically offers better medical coverage, benefits, and pay, are more likely to use “high quality” healthcare services for treating both mild and serious illnesses.

We found that healthcare-seeking behaviors differ between the elderly living in urban and rural areas, but only for women with mild diseases and men who need follow-up treatment. The accessibility of healthcare resources is an important factor influencing the healthcare-seeking behaviors of the elderly. The distribution of healthcare resources in different regions and the difference between urban and rural healthcare resources affects and restricts an individual’s healthcare provider preference. The distribution of medical resources affects the convenience of obtaining high-quality healthcare resources for the elderly. According to the Shanghai Municipal Health Commission’s public report on tertiary hospitals in Shanghai, by 2018, more than 60% of the tertiary hospitals were distributed in the central urban area, while 20.8% and 13.2% of such hospitals were located in the near and far suburbs, respectively.

Regarding medical security status, the data show that the Female elderly with basic medical insurance prefer high-grade hospitals for follow-up treatment of serious illnesses. The role of medical insurance seems not so important in daily medical treatment. The reimbursement ratio of medical insurance varies according to the level of the medical institutions, for instance, the lower the hospital level, the higher the reimbursement ratio and the lower the proportion of self-financed services.

Healthcare-seeking behaviors are determined by a complex interplay among a variety of characteristics. These include many patient characteristics as well as several structural, process, and outcome characteristics of the providers [47]. In this context, this study has some limitations. First, some factors that may be linked to individuals’ preferences have not been included in this study, such as the amount of knowledge individuals have acquired about their conditions and their previous healthcare experiences. Among individuals’ interactions and experiences with health professionals, the doctor-patient relationship particularly influences healthcare-seeking behaviors [48, 49]. Future studies should take these aspects into account. Second, the influencing factors included in the models may provide insufficient explanations for the gender differences in the elderly population’s healthcare-seeking behaviors. For instance, because many of our respondents had retired, occupation and income factors may not truly reflect their current socioeconomic characteristics. Paying more attention to the long-term cumulative effects of these factors would be helpful. Third, health conditions were measured based on self-assessment data, which might be affected by the elderly population’s physiological and psychological conditions. Finally, the samples outside Shanghai, which is indeed a new perspective that we can study in the future.

## Conclusion

The study may support some policy recommendations. On the one hand, based on the fact that income affects the elderly to seek medical services, this paper proposes that we should consider providing more convenient medical care services to the low-income elderly in an appropriate way. Medical policy support, especially for low-income elderly people, may be an important way to reduce the gap in access to medical services. On the other hand, we should pay attention to the gender differences in the elderly’s choice of medical treatment behavior, and consider the differences in the needs of male and female elderly. Reduce the inequity of medical treatment caused by differences. Just as the basic medical insurance plan in Shanghai does not play a universal role, we should continue to strengthen the design of the medical insurance system, guide and encourage the elderly to use medical services reasonably, and reasonably select the place of medical treatment according to the severity of the disease.

## Data Availability

The datasets used and/or analysed during the current study are available from the corresponding author on reasonable request.
